# Knowledge on hypertension in Myanmar: levels and groups at risk

**DOI:** 10.12688/openreseurope.14415.1

**Published:** 2022-01-28

**Authors:** Zinzi E. Pardoel, Robert Lensink, Maarten Postma, Hla Hla Win, Khin Hnin Swe, Claire Stein, Ratih Febrinasari, Hoang My Hanh, Jaap A.R. Koot, Johanna A. Landsman, Sijmen A. Reijneveld

**Affiliations:** 1 Health Sciences, University Medical Center Groningen, Groningen, Groningen, 9700 RB, The Netherlands; 2 Faculty of Economics and Business, University of Groningen, Groningen, Groningen, The Netherlands; 3 University of Public Health, Yangon, Myanmar; 4 HelpAge International, Yangon, Myanmar; 5 Department of Pharmacology, Universitas Sebelas Maret, Surakarta, Indonesia; 6 Health Strategy and Policy Institute, Hanoi, Vietnam

**Keywords:** Community health, Community-member, health literacy, Non-communicable diseases, health promotion, knowledge, hypertension, health behaviour

## Abstract

**Background: **Non-communicable diseases, specifically the burden of hypertension, have become a major public health threat to low- and middle-income countries, such as Myanmar. Inadequate knowledge of hypertension and its management among people may hinder its effective prevention and treatment with some groups at particular increased risks, but evidence on this is lacking for Myanmar. The aims of this study were therefore to assess the level of knowledge of risk factors, symptoms and complications of hypertension, by hypertension treatment status, community group-membership, and sociodemographic and socioeconomic factors in Myanmar.

**Methods: **Data was collected through structured questionnaires in 2020 on a random sample of 660 participants, stratified by region and existence of community groups. Knowledge of hypertension was measured with the ‘Knowledge’ part of a validated ‘Knowledge, Attitude and Practice’ survey questionnaire and categorised into ill-informed and reasonably to well-informed about hypertension. **Results: **The majority of respondents were reasonably to well-informed about risk factors, symptoms and complications of hypertension. This did not vary by hypertension treatment status and community group membership. People with jobs (B=0.96; 95%-confidence interval 0.343 to 1.572) and higher education (B=1.96; 0.060 to 3.868) had more hypertension knowledge than people without jobs or low education. Adherence to treatment among hypertensive people was low.

**Conclusion: **This study shows a majority of participants in Myanmar to be reasonably to well-informed, with no differences by hypertension status, treatment status, and community group-membership. People without jobs and low education have less hypertension knowledge and almost half of the hypertensive patients did not take their medicines, making them priority groups for tailored education on health care level as well as community level, lowering the burden of hypertension. Therefore, adherence to treatment of hypertension should be an important element for future health education.

## Plain language summary

Non-communicable diseases, specifically the burden of hypertension, have become a major public health threat to low- and middle-income countries, such as Myanmar. Inadequate knowledge of hypertension and its management among people may hinder its effective prevention and treatment. Socioeconomic and demographic factors, such as age and gender, have been suggested influencing level of knowledge, but evidence on this assumption lacks for Myanmar. Based on the findings in this research a training programme will be designed for community program volunteers and primary healthcare workers in Myanmar with special attention for informing vulnerable group members: people with low socioeconomic status and low health literacy. In the training programme, hypertension as chronic disease will be emphasised. In addition, the importance of adherence to treatment and how to influence that, will be high on the agenda. A cultural and contextual sensitive approach will be adopted for training in the community program, taking cognisance of local beliefs and customs. Health workers will be trained in motivational interviewing and counselling. Implementing these activities in the community programs, combined with training of healthcare staff, could lead to health gains and better cooperation and synergy between healthcare and community programs.

## Introduction

Non-communicable diseases (NCDs) are the leading cause of morbidity and mortality worldwide, also in low- and middle-income countries (LMIC), such as Myanmar ^1^. Myanmar is a country in transition with sociodemographic changes, urbanisation and industrialisation ^2, 3^. In Myanmar, unhealthy behaviour is becoming more common, *i.e.* smoking, eating processed foods, and a sedentary lifestyle, which are risk factors for developing NCDs ^4^. Specifically, the burden of hypertension has become a major public health issue ^5, 6^. In Myanmar, in 2016, high blood pressure among adults was found in 22% of females and 23% aged 15 years and older ^7^.

 Healthy behaviour contributes to prevention and control of hypertension and other NCDs, and knowledge is one of the preconditions for healthy behaviour ^8^. According to the Theory of Reasoned Action knowledge is complemented by intentions, attitudes, subjective norms and beliefs, leading to healthy behaviour ^9^. Understanding the determinants of health behaviours, *e.g.* as described by the Theory of Reasoned Action ^9^, may provide insights for targeting prevention. According to this theory, behaviour is predicted best by intentions, attitudes, subjective norms and beliefs. In turn, attitudes and subjective norms are established by behavioural, normative and control beliefs, which are based on knowledge. Critical health literacy, *i.e.* the capacity to obtain, process and understand basic health information and services needed to make appropriate health decisions, determines how well people are able to translate knowledge into appropriate (health-) behaviour ^10, 11^. Furthermore, knowledge of diseases and risk factors is influenced by socio-demographic factors ^12– 15^. Higher socioeconomic status and younger age are associated with a higher level of knowledge ^16, 17^. Having hypertension is also associated with improved knowledge of the disease ^18– 20^. In addition, if patients are aware of medication-use, physical activity and diet, they a more able to self-manage and prevent complications. It is also known that communication between physicians and hypertensive patients can improve knowledge of treatment and management of hypertension ^21^. Moreover, diagnosis of a disease leads to health seeking behaviour, i.e. searching for additional information and help beyond the medical consultation ^22^.

 To date, research about factors that influence the level of knowledge about hypertension is scarce in Myanmar. In order to set priorities for strengthening knowledge of hypertension in Myanmar, this explorative study assesses: 1) the level of knowledge of risk factors, symptoms and complications of hypertension and 2) whether this knowledge differs between hypertensive patients and non-patients and between community group-members or non-members, and by sociodemographic and socioeconomic factors. 

 The study also looks specifically at Inclusive Self-help Groups (ISHGs), which are community-based groups, in which community members support each other in improving livelihoods, social welfare, protection, health and care ^23^. There are over 150 of such groups in Myanmar and the research project Scaling-Up NCD Interventions in South East Asia (SUNI-SEA) investigates possibilities to strengthen prevention of hypertension and diabetes through these groups ^24^. Understanding hypertension knowledge among community group-members can help adapt interventions in these groups in order to increase effective prevention and control of hypertension.

## Methods

### Study design and sample

We analysed cross-sectional survey data collected in Myanmar (n=660), from community group-members (n=102) and non-members (n=558). The survey was conducted before the recent political changes. The sampling method was a stratified random sampling in three regions: Yangon (nine ISHGs), Mandalay (32 ISHGs) and Ayeyarwaddy (34 ISHGs). Per region, two villages, one with ISHGs and one without ISHGs, were included. Geographically, the Yangon region townships were urban, and the townships in Ayeyarwady and Mandalay were rural. Per village, 55 participants were included, aged 40 years and older, resulting in 220 participants per region and 660 participants in total. In villages with an ISHG, both members and non-members were included. The study population included both males and females, residing in the study area for at least six months.

### Procedure

Data were collected in January 2020 door-by-door, by interviewers using tablets with data collection and processing software KoBo ^25^. Trained enumerators collected data using structured questionnaires, including questions on lifestyle factors, knowledge and perception on hypertension, practices related to hypertension, and accessibility to healthcare services provided by ISHGs. The interviews started with open questions per topic. If the participant did not provide an answer, data collectors started probing, *i.e.* asking related questions, leading to better understanding of the questions and more relevant answers. Participation in the surveys was voluntary and informed consent was obtained from all participants before any data was collected.

### Measures

*Background, sociodemographic and socioeconomic variables* regarded *age*, which we recoded into younger (=0) and older (=1) using the median as cut-off point and *gender (male=0 and female=1)*, *marital status* (not married=0 and married=1), *job status* (not-working=0 and working=1) and *living area* (rural=0 or urban=1). *Education level* was measured by asking the participants to indicate what the highest level of completed schooling was. Answers were categorised into low/no education (=0), consisting of no formal schooling, primary school level, secondary school level and high school level and high education (=1) consisting of college/university level and graduate.

**Figure 1.**
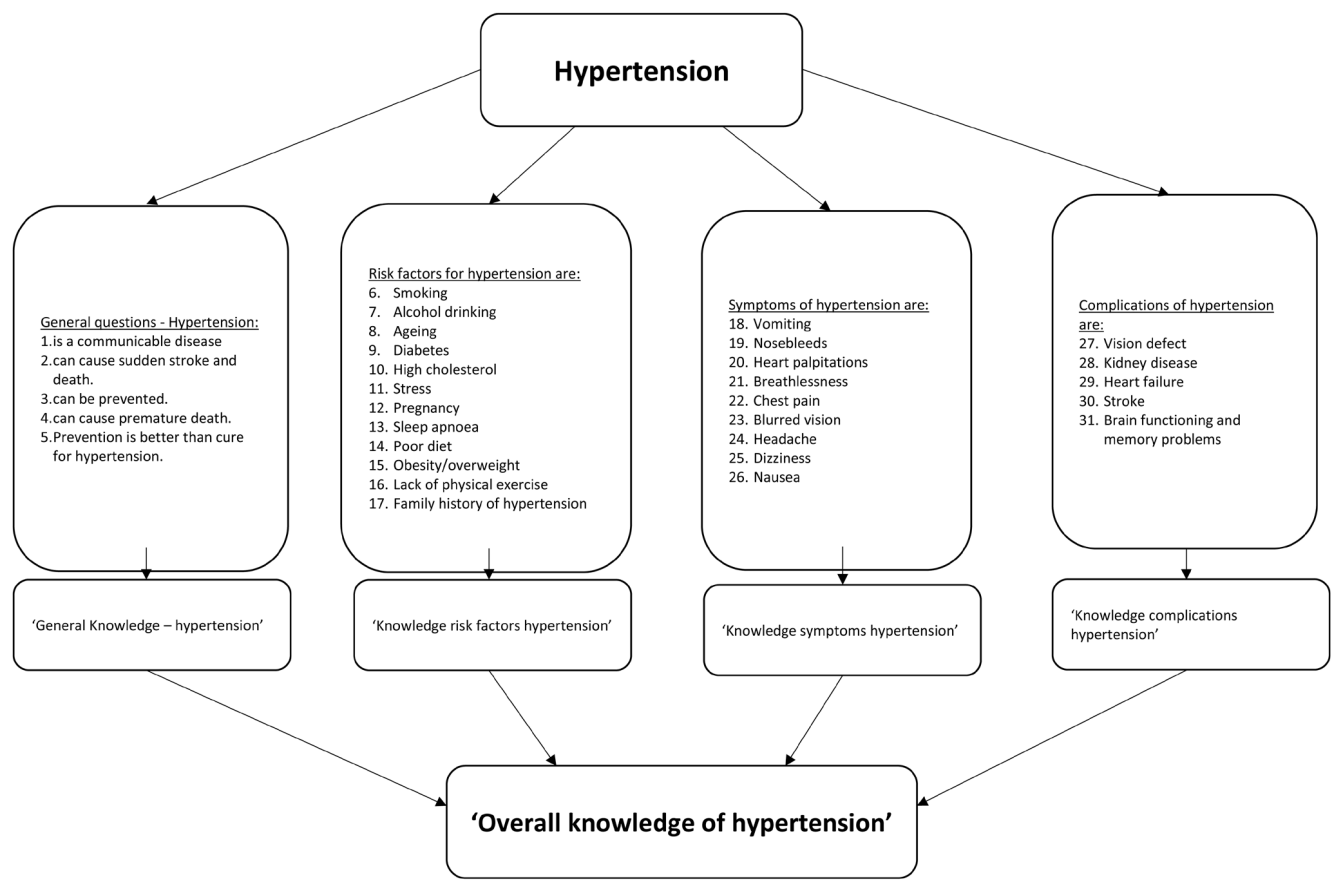
Illustration of recoding and development of knowledge variable.

*Knowledge of hypertension* was measured with the ‘Knowledge’ part of a validated ‘Knowledge, Attitude and Practice’ questionnaire ^26^ (see the Extended Data for all knowledge questions). Respondents were asked to indicate whether an item concerning general knowledge of hypertension, risk factors, symptoms and complications was ‘true’ or ‘false’. For example, ‘risk factors for hypertension are the following: smoking, alcohol drinking, ageing, diabetes, high cholesterol, stress, pregnancy, sleep apnoea, poor diet, obesity or overweight, lack of physical exercise and family history of hypertension’. Knowledge was measured on four topics (see Figure 1 for the items per topic): general knowledge, risk factors, symptoms, and complications. All items (n=31) could be answered with true (=1) or false/do not know (=0). All items together created an overall knowledge variable for hypertension. Cut-offs for knowledge level were derived from similar other studies ^27, 28^, namely: reasonably to well-informed (number of correct answers ≥ mean) or ill-informed (else). For six items, the percentage missing was higher than 10% ^29^. To obtain approximately unbiased estimates of all parameters, we performed multiple imputation, based on all variables used for the analysis.

*Hypertension* was measured by asking participants if they had hypertension (self-reported) and if they took medicines for hypertension. This was categorised as hypertensive patients with treatment (prescribed medicine), hypertensive patients without treatment and non-patients. Moreover, hypertensive patients indicating not taking prescribed medicine were asked to give reasons for not taking medicine in an open question. *Community group-membership* was asked straightforwardly (no=0 or yes=1).

### Analysis

First, we described the background characteristics of the sample. Second, we assessed the level of knowledge for risk factors, symptoms and complications. Third, we explored if hypertension treatment status, community group-membership, sociodemographic and socioeconomic factors were significantly associated with level of knowledge, using linear regression analyses, crude and mutually adjusted. To minimise potential bias, analyses were performed with the multiple imputed dataset, consisting of 10 data imputations, based on the assumption that the data was missing at random. In the imputed dataset, *e.g.* a pooled dataset based on 10 different plausible imputed datasets, all cases are preserved by replacing missing data with an estimated value based on the available information. A p value of <0.05 (two-tailed) was considered statistically significant for all associations. All measurements and analysis were carried out with IBM SPSS Statistics 26.

## Results

### Background characteristics

Table 1 shows the results of the descriptive analysis of the background characteristics of the sample population. In total, 179 (27%) men were included in the study with an average age of 57.5 (±10.7) years. Most respondents had a low education level (97%), lived in a rural area (62%), were married (71%), indicated no community group-membership (84%) and were working (76%). Forty-five percent reported that they had hypertension and 175 respondents indicated taking medicine for hypertension. The most mentioned reasons by hypertensive patients for not taking medicines were ‘ *feeling better’* and ‘ *not having access to medication’*.

**Table 1.** Baseline characteristics of study population.

Characteristics study population (n=660)	Categories	n (%)
**Gender**	*Male*	179 (27)
	*Female*	481 (73)
**Educational level ^i^**	*Low education*	638 (97)
	*High education*	21 (3)
**Living area**	*Rural*	406 (62)
	*Urban*	254 (38)
**Marital status ^ii^**	*Married*	470 (71)
	*Not married*	188 (29)
**Community group-members**	*Member*	102 (16)
	*Non- member*	558 (84)
**Job status**	*Working*	501 (76)
	*Non-working*	159 (24)
**Self-reported hypertension**	*No*	364 (55)
	*Yes*	296 (45)
**Taking medicine for hypertension ^iii^**	*No*	121 (41)
	*Yes*	175 (59)

^i^ Missing = one person refused to answer ^ii^ Missing = two persons refused to answer ^iii^ Out of those with self-reported hypertension (n=296)

### Level of knowledge of risk factors, symptoms and complications for hypertension

Table 2 shows rates of knowledge about hypertension for the total group and separately for hypertensive patients with treatment, patients without treatment, non-patients, community group-members and non-members. The majority of participants are reasonably to well-informed about hypertension. The proportion of reasonably to well-informed participants is highest among the group patients with treatment (91.4%) and lowest in the non-patients’ group (86.5%).

**Table 2.** Distribution of ill-informed and reasonably to well-informed about hypertension among patients, non-patients, group-members and non-members.

Level of knowledge	All n (%)	Patients with treatment n (%)	Patients without treatment n (%)	Non-patients n (%)	Community group-members n (%)	Non-members n (%)
**Mean ± stdev ^i^**	19.7±5.8	20.0±5.3	20.3±5.7	19.3±6.1	19.7±5.7	19.7±5.8
*Ill-informed*	78 (11.8)	15 (8.6)	13 (10.7)	49 (13.5)	11 (10.8)	66 (11.8)
*Reasonably to well-informed*	582 (88.2)	160 (91.4)	108 (89.3)	315 (86.5)	91 (89.2)	492 (88.2)
**Total**	660 (100)	175 (100)	121 (100)	364 (100)	102 (100)	558 (100)

^i^Mean and standard deviation of all knowledge items

### Differences in the level of knowledge for hypertensive patients, community group-members and sociodemographic and socioeconomic factors

Hypertensive patients with and without treatment scored highest on knowledge of symptoms and complications. Community group-members scored lowest on knowledge of symptoms. Community group-members scored highest on knowledge of risk factors and non-patients the lowest without significant differences among groups.

 The results of the linear regression analysis of the association of level of knowledge with sociodemographic and socioeconomic factors, hypertensive patients with treatment and without treatment and community group-members is shown in Table 3, in which model 1 shows results for crude and model 2 for mutually adjusted analyses. In model 1 three sociodemographic and socioeconomic factors were associated with the level of knowledge, *i.e.*, job status (B=1.07; 95%-confidence interval 0.505 to 1.631), educational level (B=2.14; 0.428 to 3.854) and age (B=-1.08; -1.991 to -0.162). This indicates that employed, higher educated and younger people have more hypertension knowledge than unemployed, lower educated and older people. In the adjusted model 2, job status (B=0.96; 0.343 to 1.572) and educational level (B=1.96; 0.060 to 3.868) were associated with level of hypertension knowledge, whereas age was not.

**Table 3.** Differences in level of: results of regression analyses leading to regression coefficients (B) and 95%-confidence intervals.

Socio-demographic factors	Model 1 ^a^ B(95% CI)	Model 2 ^b^ B(95% CI)
Marital status (married vs. not married)	-0.47(-1.473;0.540)	0.33(-0.869;1.530)
Educational level (high vs. low)	2.14(0.428;3.854) **	1.96(0.060;3.868) *
Gender (female vs. male)	0.12(-0.923;1.160)	-0.46(-1.653;0.730)
Living area (urban vs. rural)	-0.30(-1.285;0.695)	-0.67(-1.847;0.510)
Age (old vs. young)	-1.08(-1.991;-0.162) *	-0.69(-1.827;0.452)
Job-status (working vs not-working)	1.07(0.505;1.631) **	0.95(0.275;1.629) **
**Hypertensive and community group-member**		
Patients with treatment	0.73(-0.340;1.799)	1.06(-0.046;2.170)
Patients without treatment	1.12(-0.127;2.356)	0.97(-0.272;2.206)
Community group-members	0.01(-1.241;1.262)	-0.10(-1.575;1.368)

^a^ Crude variables analyses ^b^ Variables mutually adjusted analysis ^*^ Significant by p<0.05 ^**^ significant by p<0.01

 No significant associations were found in the knowledge scores between hypertensive patients with or without treatment or non-patients. In addition, no significant differences were found for community group-members or non-members. 

## Discussion

We found that the majority of respondents seem reasonably to well-informed of risk factors, symptoms and complications regarding hypertension. No differences were found in level of knowledge between hypertension patients with or without treatment and non-patients and between community group-members and non-members. Employment and higher education level were positively associated with the level of hypertension knowledge. 

 We found that the majority of participants in the survey were reasonably to well-informed about hypertension, which contrasts with findings of similar studies on hypertension knowledge in other Asian countries that show mainly poor hypertension knowledge ^30, 31^. A possible explanation for this finding could be the adoption of the WHO Package of Essential Non-Communicable (PEN) Diseases Interventions in Myanmar ^32^. As part of the National Strategic Plan for Prevention and Control of NCDs (2017-2021) ^33^ the PEN was introduced in the whole country, aiming to strengthen health systems including health education among healthcare staff and citizens. The publicity around the PEN could have led to an improvement of hypertension knowledge in Myanmar.

 We found no differences in knowledge between hypertensive patients and non-patients, and neither between patients that took hypertension medication and patients that did not. This contrasts with evidence from other research were patients showed to have more knowledge about their illness ^34^. This may be explained by insufficient communication between health workers and patients in the research areas in this study in Myanmar ^35– 38^. It is known that the capacities of human resources for health in Myanmar are limited ^39^. The training of health workers is not covering NCDs as a topic, and only since the introduction of the PEN programme on-the-job training was introduced. In rural areas in Myanmar, healthcare coverage is inadequate and healthcare workers do not have time for communication about NCDs and risk factors ^39^.

 Furthermore, we found that over 40% of the hypertensive patients reported not taking their prescribed medicines. Non-adherence to antihypertensive drugs can have adverse health outcomes, such as stroke and kidney damage ^40, 41^. Several factors may contribute to non-adherence. First, patients may not feel the urgency to adhere to treatment for a longer period of time ^42^. Patients show poor treatment follow-up even after the initial consultation ^32^. Long travelling times or unavailability of medicine may contribute to poor adherence ^32^.

 Community Inclusive Self-help Groups (ISHGs) members did not have better knowledge. This is not in line with previous research, which showed health promotion activities in community-based programs being effective in improving health-related knowledge and healthy behaviour ^43– 45^. An explanation for this finding may be that the community groups had not yet undertaken as many health activities in relation to prevention of NCDs.

 Finally, we found that job-status and educational level were associated with the level of knowledge, which confirms evidence from other LMIC ^15, 16^. People with a lower socioeconomic status have a lower level of hypertension knowledge, which makes them more vulnerable to hypertension. Low educational level is also associated with limited health literacy ^46^, which can result in disadvantageous health outcomes.

### Strengths and limitations

Strengths of this study are the comprehensiveness of the data that was collected, including multiple sociodemographic and socioeconomic factors, variables about hypertension knowledge, covering different geographical areas in Myanmar. Another strength is the stratified random sampling method, which ensured each area was adequately represented within the whole sample population of this study.

 A limitation of this study is the unknown response rates, combined with a reasonable number of missing values. We solved this by multiple imputation, creating several different plausible imputed datasets and combining the results. Another limitation of the present study is the method of measuring knowledge of hypertension in the questionnaire. All items for general knowledge, risk factors, symptoms and complications were positively formulated, which is commonly done in KAP-surveys. This could have resulted in response bias. Moreover, this is a relatively small size study, which may have resulted in finding mainly the strongest associations. Even with these limitations, this is one of the few studies, to our knowledge, that report on the level of hypertension knowledge and differences in hypertension knowledge between hypertensive patients, non-patients, community group-members and non-members in Myanmar.

### Implications

The research was carried out in the context of project ‘Scaling Up Non-communicable diseases Interventions in South East Asia’ (SUNI SEA; 2019-2022). Based on the findings in this research a training programme will be designed for ISHG volunteers and primary healthcare workers. There will be special attention for informing vulnerable group members: people with low socioeconomic status and low health literacy. In the training programme, hypertension as chronic disease will be emphasised. In addition, the importance of adherence to treatment will be high on the agenda. A cultural and contextual sensitive approach will be adopted for training ISHGs, taking cognisance of local beliefs and customs. Health workers will be trained in motivational interviewing and counselling. Implementing these activities in the ISHGs, in combination of training healthcare staff, could lead to health gains and better cooperation and synergy between healthcare and community programs ^43– 45^.

 The findings of this study require conformation in a larger study. With improved data collection tools, sensitivity and power problems can be limited. Follow-up studies after training ISHGs are planned in the SUNI-SEA project, which could measure differences in knowledge as result of the training.

## Conclusion

This study showed that the majority of participants in the study in Myanmar seem reasonably to well-informed of risk factors, symptoms and complications concerning hypertension, with no differences between hypertensive patients and non-patients, and no differences between community group-members and non-members. Unemployed and lower educated people have less hypertension knowledge, indicating them as priority groups for health education. Increasing knowledge of hypertension and its management among people will enhance prevention and treatment. This study guides the interventions in this regard in Myanmar. Thus, we suggest implementing health-related activities at community level and improving health-related education at the primary healthcare level. In order to effectively lower the burden of hypertension, synergies between community programmes and primary healthcare should be implemented.

## List of abbreviations

e.g.: exempli gratia, Latin phrase meaning "for example"

HL: health literacy

FHL: functional health literacy

i.e.: id est, Latin phrase meaning "that is".

ISHG: Inclusive Self-help Groups

NCD: non-communicable disease

## Data availability

### Underlying data

**Zenodo: Underlying data for ‘Knowledge on hypertension in Myanmar: levels and groups at risk’. https://doi.org/10.5281/zenodo.5880978**

### Extended data

**Zenodo: Extended data for ‘Knowledge on hypertension in Myanmar: levels and groups at risk’. https://doi.org/10.5281/zenodo.5881186**

### Reporting guidelines

**Zenodo: STROBE checklist for ‘Knowledge on hypertension in Myanmar: levels and groups at risk’. https://doi.org/10.5281/zenodo.5881253**

Data are available under the terms of the Creative Commons Attribution 4.0 International license (CC-BY 4.0).

### Ethics and consent

Ethics approval for the primary study was obtained from the Institutional Review Board (IRB) of the Department of Medical Research (DMR) of the Ministry of Health in Myanmar, under IRB number 2019-137 and approval number Ethics/DMR/2019/145.

All patients gave written and oral informed consent to participate in the study before taking part. 
