# Supplementary material for: Knowledge on hypertension in Myanmar: levels and groups at risk
Source: Open Res Eur. 2023 Mar 21;2:13. Originally published 2022 Jan 28. [Version 2] doi: 10.12688/openreseurope.14415.2 (PMC10445853; doi:10.12688/openreseurope.14415.2)
Supplement: Peer review history

# Peer review: Reviewer response for version 2

**DOI:** 10.21956/openreseurope.17042.r31030

**Published:** 2023-04-04

**Authors:** Martin Heine

**Affiliations:** 1 Department of Global Public Health & Bioethics, Julius Centre for Health Sciences and Primary Care, University Medical Centre Utrecht, Utrecht, The Netherlands

Thank you for addressing the comments provided, and congratulations on an important study for the management and prevention of HTN in Myanmar.

 For future research, you could consider developing more adequate cut-off value for HTN knowledge in relation to risk or clinical outcomes as I do feel that the current dichotomisation is still a bit unsatisfactory; however, this is also clearly mentioned now in the limitations section.

Is the study design appropriate and does the work have academic merit?

Yes

Is the work clearly and accurately presented and does it cite the current literature?

Yes

If applicable, is the statistical analysis and its interpretation appropriate?

Partly

Are all the source data underlying the results available to ensure full reproducibility?

Yes

Are the conclusions drawn adequately supported by the results?

Partly

Are sufficient details of methods and analysis provided to allow replication by others?

Partly

Reviewer Expertise:

non-communicable disease, global health, implementation science

I confirm that I have read this submission and believe that I have an appropriate level of expertise to confirm that it is of an acceptable scientific standard.

# Peer review: Reviewer response for version 1

**DOI:** 10.21956/openreseurope.15559.r30796

**Published:** 2023-02-06

**Authors:** Martin Heine

**Affiliations:** 1 Department of Global Public Health & Bioethics, Julius Centre for Health Sciences and Primary Care, University Medical Centre Utrecht, Utrecht, The Netherlands

**Introduction:**

 There are many theories that aim to explain behavior, of which the Theory of reasoned action is one. It is not clear why specifically this theory was adopted for understanding prevention and control of HTN in Myanmar; specifically, as this theory largely “excludes” eccentric/environmental factors that inform behavior. For example, we have shown that for physical activity, a more holistic approach would be needed to fully grasp PA behavior when settings with limited resources are concerned (Heine *et al.*, 2021 ^1^). A framework of behaviour that is more response to contextual factors, may also aid the introduction (and selection) of the variables of interest you specifically looked at (e.g., socio-economic status).

 In the second to last paragraph you mention that you will look at “community-members” and non-members, however, at that stage, the concept of the ISHG’s has not been introduced, hence, it is not clear what “community” you refer to.

**Methods:**

Study settings and sample:

- Would you be able to provide a bit more detail on the context for each village or region? This will be helpful in interpreting the findings. For example, the CICI framework (Pfadenhauer *et al.*, 2017 ^2^) provides an overview of different contextual factors one could consider; and also our group (van Zyl *et al.*, 2021 ^3^) has provided some guidance and discussion as to the resources often challenged in low-resourced settings, and argue for a more in-depth interrogation on these when reporting study findings.

Procedure:

- Please provide detail on ethical approval.

Measures:

- Please provide a rationale for using the “median” as a way to dichotomize age? Given the mean age, would you really argue that someone e.g., 56 is a “younger” adult? Is there no better way of doing this (e.g., based on burden of disease, or more sub-groups)?
- Same for education level; is there a reason to dichotomize? Seems to me you lose nuances by doing so, e.g. higher education, in any country, would be a significant education upbringing, like to affect health seek-behaviour, disease knowledge etc. - this is now grouped with no education.

Hypertension:

- Ideally, one would also have collected objective measures for HTN, being the primary outcome. Working in South African low-resourced settings, we noticed for instance that quite a number of participants have been diagnosed with hypertension yet were unaware, or were receiving HTN medication, however could not explain what the medication they were taking was for (in particular in uneducated participants). Hence, this will impact validity of self-reported measures of morbidity (in this case, hypertension). I would argue this should be included in the limitations section.

Analysis:

- Wouldn’t this be considered logistic regression, seeing that you dichotomized the hypertension knowledge outcome?
- Dichotomization of HTN should be reconsidered. If all participants have very poor knowledge, then the mean score would be very low - having scored higher than the mean score, should then not lead to a conclusion that that specific participant is reasonably- to well-informed. Are there no validated cut-off points for this knowledge measure? Alternatively, one could either use IQRs, or treat the outcome as a continuous score between 0 – 31.
- Could you provide a bit more detail on the regression analyses done? I assume the crude analysis, is univariate? And the mutually adjusted is a multivariate analysis? Did you plan to include all variables in the adjusted analysis? Or just the ones that reach significant or explained a certain percentage of variance? etc. It is difficult to interpret your findings in this way.

**Results:**

- Please see earlier comment on grouping of education level. A 97% low-education is not surprising given your definition, plus, within the context of Myanmar, is e.g., having completed high school or higher education not considered quite an achievement (i.e. is this really “low” education, within the context of Myanmar?). I would really argue for stratifying the analysis in addition to subgroups for education, and using no formal education as a reference group (possibly in conjunction with those not completed elementary school). One could also look at a national census (if available for Myanmar) to see how they grouped education levels.
- Given the random sampling technique, can you explain the vast difference in male and female participation? This suggests selection bias, and should be acknowledged in the limitations section. It is also interesting in itself in relation to the prevention/treatment of HTN. What were the factors leading to the higher female inclusion (e.g., men at work, etc)?
- In your figure, detailing the various questions you highlight four “sub-scales”; is there value in presenting data for those as well? For instance, I would argue that it is less problematic if someone without hypertension knows about HTN complications, and its more important to know about risk factors? This could be an avenue to be explored to provide more depth to your analyses.

**Discussion:**

- Based on the analysis currently provided, the “main finding” presented in the first paragraph of the discussion seems the artefact of definition, rather than a “true” finding. E.g., 50% of your sample (assuming normal distribution) scored less than 19.7 out of 31.

Is the study design appropriate and does the work have academic merit?

Yes

Is the work clearly and accurately presented and does it cite the current literature?

Yes

If applicable, is the statistical analysis and its interpretation appropriate?

Partly

Are all the source data underlying the results available to ensure full reproducibility?

Yes

Are the conclusions drawn adequately supported by the results?

Partly

Are sufficient details of methods and analysis provided to allow replication by others?

Partly

Reviewer Expertise:

non-communicable disease, global health, implementation science

I confirm that I have read this submission and believe that I have an appropriate level of expertise to confirm that it is of an acceptable scientific standard, however I have significant reservations, as outlined above.

## References

[1] : Developing a Complex Understanding of Physical Activity in Cardiometabolic Disease from Low-to-Middle-Income Countries-A Qualitative Systematic Review with Meta-Synthesis. *Int J Environ Res Public Health* .2021;18(22) : 10.3390/ijerph182211977 10.3390/ijerph182211977 34831732PMC8619369

[2] : Making sense of complexity in context and implementation: the Context and Implementation of Complex Interventions (CICI) framework. *Implement Sci* .2017;12(1) : 10.1186/s13012-017-0552-5 21 10.1186/s13012-017-0552-5 28202031PMC5312531

[3] : Unravelling 'low-resource settings': a systematic scoping review with qualitative content analysis. *BMJ Glob Health* .2021;6(6) : 10.1136/bmjgh-2021-005190 10.1136/bmjgh-2021-005190 34083239PMC8183220

# Response

**Published:** 2023-03-13

**Authors:** Zinzi Pardoel

**Affiliations:** University Medical Center Groningen, The Netherlands

Thank you for your comprehensive review and detailed comments. We have clarified and revised the manuscript based on your comments. Please find a detailed response, preceded by [Response] to all comments.  

**Introduction:**

 1. There are many theories that aim to explain behavior, of which the Theory of reasoned action is one. It is not clear why specifically this theory was adopted for understanding prevention and control of HTN in Myanmar; specifically, as this theory largely “excludes” eccentric/environmental factors that inform behavior. For example, we have shown that for physical activity, a more holistic approach would be needed to fully grasp PA behavior when settings with limited resources are concerned (Heine et al., 20211). A framework of behaviour that is more response to contextual factors, may also aid the introduction (and selection) of the variables of interest you specifically looked at (e.g., socio-economic status).

 [Response 1] Thank you for your suggestion. We agree and have now introduced another framework of behaviour, i.e., the socio-ecological model, in the introduction. The revised text is (new text has been italicized): *“According to the socioecological model, health behaviour is also determined by  the social, physical and policy environment in which it occurs* *[1]* *. Socio-demographic factors influence the knowledge of diseases and risk factors, therefore behaviour.”* *[* *1] Stokols D. Translating social ecological theory into guidelines for community health  promotion. Am J Health Promot. 1996;10(4):282–98.* *[2].* *Heine, M., Badenhorst, M., van Zyl, C., de Melo Ghisi, G. L., Babu, A. S., Buckley, J., … Derman, W. (2021). Developing a Complex Understanding of Physical Activity in Cardiometabolic Disease from Low-to-Middle-Income Countries—A Qualitative Systematic Review with Meta-Synthesis. International Journal of Environmental Research and Public Health, 18(22). https://doi.org/10.3390/ijerph182211977* 2.

 In the second to last paragraph you mention that you will look at “community-members” and non-members, however, at that stage, the concept of the ISHG’s has not been introduced, hence, it is not clear what “community” you refer to.

 [Response 2] Thank you for comment. We have clarified this by revising the last paragraph and rearranging the order of the paragraphs. The last paragraph is now the second last paragraph. The revised text is (new text has been italicized): *“In Myanmar, community-based groups, known as Inclusive Self-help Groups (ISHGs) are aimed to improve the wellbeing of vulnerable groups, such as people at risk for hypertension or with hypertension.* Community members support each other in improving livelihoods, social welfare, protection, health and care.”

**Methods:** Study settings and sample: 3.  Would you be able to provide a bit more detail on the context for each village or region? This will be helpful in interpreting the findings. For example, the CICI framework (Pfadenhauer et al., 20172) provides an overview of different contextual factors one could consider; and also our group (van Zyl et al., 20213) has provided some guidance and discussion as to the resources often challenged in low-resourced settings, and argue for a more in-depth interrogation on these when reporting study findings.

 [Response 3] We have added some more contextual information about the regions to the Methods section, namely: *“All three regions are located in the centre of Myanmar. The Yangon region is most populated, with over seven million people. It is an ethnically diverse region, with different religions and the primary economical source of livelihood is trade. The Mandalay region is the second most populated region, with around 6.2 million people and over 95% of the population being Buddhists and with agriculture being the primary source of livelihood. The Ayeryarwady region is the most rural region, with 6.5 million people and 92% of the population being Buddhists. Agriculture is the main source of livelihoods.”*

 Procedure: 4.  Please provide detail on ethical approval.

 [Reponse 4] We have added the information on the ethical approval of the primary data collection to the procedure, based on your suggestion, namely: *“Ethics approval for the primary study was obtained from the Institutional Review Board (IRB) of the Department of Medical Research (DMR) of the Ministry of Health in Myanmar (IRB number 2019-137 and approval number Ethics/DMR/2019/145).”*

 Measures: 5.  Please provide a rationale for using the “median” as a way to dichotomize age? Given the mean age, would you really argue that someone e.g., 56 is a “younger” adult? Is there no better way of doing this (e.g., based on burden of disease, or more sub-groups)?

 [Response 5] Our rationale for using the median to dichotomize age is a skewed distribution of age (40 -87 years) and dichotomizing provides more power in the analysis. We have added text to clarify this, namely: *“and age, which we recoded into younger (=0) and older (=1) using the median as cut-off point. We dichotomized age because it had a skewed distribution and dichotomizing provides more power.”*

 6. Same for education level; is there a reason to dichotomize? Seems to me you lose nuances by doing so, e.g., higher education, in any country, would be a significant education upbringing, like to affect health seek-behaviour, disease knowledge etc. - this is now grouped with no education.

 [Response 6] Based on your suggestion, we categorized the variable into no formal education, low/middle education and high education. We have looked at the national census. We have added the following text under study design: *“Outside the main cities of Yangon and Mandalay educational opportunities are limited, with only 13% of the primary school students reaching high school. In Myanmar, 46% of women and 52% of men have attended secondary school at maximum and 10% of women and 7% of men have completed more than secondary education* *[2]* *.”* [2] *MOHS, ICF. Myanmar Demographic and Health Survey 2015-16. Myanmar Demogr Heal Surv [Internet]. 2017;1–37. Available from: https://dhsprogram.com/publications/publication-fr324-dhs-final-reports.cfm%0Ahttps://dhsprogram.com/pubs/pdf/FR324/FR324.pdf*

 7. Hypertension: Ideally, one would also have collected objective measures for HTN, being the primary outcome. Working in South African low-resourced settings, we noticed for instance that quite a number of participants have been diagnosed with hypertension yet were unaware, or were receiving HTN medication, however could not explain what the medication they were taking was for (in particular in uneducated participants). Hence, this will impact validity of self-reported measures of morbidity (in this case, hypertension). I would argue this should be included in the limitations section.

 [Response 7] We agree that self-reported hypertension can impact the validity of measuring. We have added a comment on potential information bias due to self-reported hypertension and medication use to the limitations part of the Discussion section, namely: “ *Furthermore, in this study we used self-reported hypertension and use of medication for hypertension. Inadequate self-reporting could have led to underestimating the actual number of hypertensive patients and the medication adherence*.”

 Analysis: 8. Wouldn’t this be considered logistic regression, seeing that you dichotomized the hypertension knowledge outcome?

 [Response 8] We did our analyses with use of hypertension knowledge as a continuous variable. We therefore used a linear regression. We have provided some additional information to clarify this in the Methods section. The revised text is (new text has been italicized): “ *A continuous variable on hypertension knowledge was created including scores from 0 to 31. A dichotomized variable was created based on cut-offs* for knowledge level derived from similar other studies ^27^^, ^^28^ , namely: reasonably to well-informed (number of correct answers ≥ mean) or ill-informed (else).” *[27] Kassahun CW, Mekonen AG: Knowledge, attitude, practices and their associated factors towards diabetes mellitus among non diabetes community members of Bale Zone administrative towns, South East Ethiopia. A cross-sectional study. PLoS One. 2017;12(2):e0170040. 28152066 10.1371/journal.pone.0170040 5289457* *[28] Parmar P, Rathod GB, Rathod S, et al.: Study of knowledge, attitude and practice of general population of Gandhinagar towards hypertension. Int J Curr Microbiol App Sci. 2014;3(8):680–685.* Moreover, we clarified our use of a continuous variable by adding the following text to the footnote of Table 3 in the Results section: “ ^a^ *The continuous variable of level of knowledge, with scores between 0-31, was included in the analyses*”

 9. Dichotomization of HTN should be reconsidered. If all participants have very poor knowledge, then the mean score would be very low - having scored higher than the mean score, should then not lead to a conclusion that that specific participant is reasonably- to well-informed. Are there no validated cut-off points for this knowledge measure? Alternatively, one could either use IQRs, or treat the outcome as a continuous score between 0 – 31.

 [Response 9] Thank you for pointing this out. First, we have based our cut-off for the knowledge level on similar studies measuring the knowledge part of the Knowledge, Attitude, and Practice-survey. In these studies ^27,28^, the mean score was used as cut-off for dichotomizing knowledge into ‘Good knowledge’ vs ‘Poor knowledge’. We have dichotomized as ‘reasonably to well-informed’ vs ‘ill-informed. The dichotomized knowledge variable is used for the describing levels of knowledge in different groups (e.g., married vs. not married).

 Second, we used the outcome as a continuous score between 0 – 31 in the analyses. See our response to your comment 8 for more clarification. [27] Kassahun CW, Mekonen AG: Knowledge, attitude, practices and their associated factors towards diabetes mellitus among non diabetes community members of Bale Zone administrative towns, South East Ethiopia. A cross-sectional study. *PLoS One.* 2017;12(2):e0170040. 28152066 10.1371/journal.pone.0170040 5289457 [28] Parmar P, Rathod GB, Rathod S, et al.: Study of knowledge, attitude and practice of general population of Gandhinagar towards hypertension. Int J Curr Microbiol App Sci. 2014;3(8):680–685.

 10. Could you provide a bit more detail on the regression analyses done? I assume the crude analysis, is univariate? And the mutually adjusted is a multivariate analysis? Did you plan to include all variables in the adjusted analysis? Or just the ones that reach significant or explained a certain percentage of variance? etc. It is difficult to interpret your findings in this way.

 [Response 10] In response to your question, we clarified this under table 3. The revised text is (new text has been italicized):  “ ^b^ Crude variables analyses: *univariate regression of hypertension knowledge and independent variable* ^c^ Variables mutually adjusted analysis: *multivariable regression of hypertension knowledge and independent variables including all potentially confounding variables included in this model.”*

**Results:** 11. Please see earlier comment on grouping of education level. A 97% low-education is not surprising given your definition, plus, within the context of Myanmar, is e.g., having completed high school or higher education not considered quite an achievement (i.e. is this really “low” education, within the context of Myanmar?). I would really argue for stratifying the analysis in addition to subgroups for education, and using no formal education as a reference group (possibly in conjunction with those not completed elementary school). One could also look at a national census (if available for Myanmar) to see how they grouped education levels.

 [Response 11] Thank you for this suggestion. We have responded to this comment at your comment 6.  

 12. Given the random sampling technique, can you explain the vast difference in male and female participation? This suggests selection bias, and should be acknowledged in the limitations section. It is also interesting in itself in relation to the prevention/treatment of HTN. What were the factors leading to the higher female inclusion (e.g., men at work, etc)?

 [Response 12] Based on your suggestion, we have added information about this selection bias in the limitation section, namely: *“Last, in this study selection bias may have occurred , taking into consideration the inclusion of a vast majority of female participants. Because data was collected during the daytime, most men were working.”*

 13. In your figure, detailing the various questions you highlight four “sub-scales”; is there value in presenting data for those as well? For instance, I would argue that it is less problematic if someone without hypertension knows about HTN complications, and its more important to know about risk factors? This could be an avenue to be explored to provide more depth to your analyses.

 [Response 13] Thank you for this suggestion. We have added results of average knowledge per subgroup as extended data. Moreover, we have added the following text: “ *The group patients with treatment scored on average highest on knowledge about risk factors for hypertension and the group of non-patients lowest (see Extended Data datafile 2 for the distribution of knowledge scores per subtopic). The group of hypertensive patients without treatment scored on average highest on knowledge about symptoms of hypertension and community group-members scored highest on general knowledge about hypertension.*” We also analysed if there were significant differences per subtopic of knowledge between hypertensive patients vs non-patients and community members vs non-members. We have added the following text in the Results section: “ *Last, no significant differences were found per subtopic of hypertension knowledge between hypertensive patients with and without treatment, and non-patients, and between community members and non-members*.”

 Discussion: 14. Based on the analysis currently provided, the “main finding” presented in the first paragraph of the discussion seems the artefact of definition, rather than a “true” finding. E.g., 50% of your sample (assuming normal distribution) scored less than 19.7 out of 31.

 [Response 14] Although the cut-off for knowledge was derived from similar studies analysing the knowledge part of the KAP-questionnaire, we agree that dichotomizing knowledge based on the mean score could have led to overestimation of knowledge levels. In Myanmar, no comparative results regarding hypertension knowledge exist and therefore no validated estimate could be drawn for our study. We have revised the sentence describing this finding namely: “ *Based on our findings, it seems that most respondents in this study were reasonably to well-informed of risk factors, symptoms and complications regarding hypertension.* *”* Moreover, we have attenuated this conclusion by revising sentences which state that respondents *were *reasonably to well-informed into respondents *seem* to be reasonably to well-informed. We also added a discussion paragraph in which we put this finding up for discussion. The new text is:   “ *Another explanation for finding that the majority of participants in this study was reasonably to well-informed about hypertension, might be that we dichotomized hypertension knowledge based on the mean score as a cut-off point. Although the cut-off was derived from similar studies, dichotomizing knowledge could have led to overestimation of knowledge levels. In Myanmar, no comparative results regarding hypertension knowledge exist and therefore no validated estimate could be drawn for our study.”*

# Peer review: Reviewer response for version 1

**DOI:** 10.21956/openreseurope.15559.r28486

**Published:** 2022-02-18

**Authors:** Pugie T. Chimberengwa

**Affiliations:** 1 Matabeleland North Province, Harare, Zimbabwe

Good article. However the authors need to relook at the abstract. The results are quoted in the conclusion and they do not tally with the overall message. Those with poor education/no jobs have a greater chance of not taking medication and this is a social grouping. This is the only bit that needs to be sorted. However, in the manuscript the information is correctly documented.

Is the study design appropriate and does the work have academic merit?

Yes

Is the work clearly and accurately presented and does it cite the current literature?

Yes

If applicable, is the statistical analysis and its interpretation appropriate?

Yes

Are all the source data underlying the results available to ensure full reproducibility?

Yes

Are the conclusions drawn adequately supported by the results?

Yes

Are sufficient details of methods and analysis provided to allow replication by others?

Yes

Reviewer Expertise:

Hypertension, NCDs and HIV/AIDS

I confirm that I have read this submission and believe that I have an appropriate level of expertise to confirm that it is of an acceptable scientific standard.

# Response

**Published:** 2023-03-13

**Authors:** Zinzi Pardoel

**Affiliations:** University Medical Center Groningen, The Netherlands

Thank you for treviewing our manuscript. We have carefully considered your comment and revised the manuscript accordingly. Please find a detailed response, preceded by [Response] to the comment. [Response] Thank you for your compliment and suggestion for revision of the abstract. We have clarified and revised the abstract based on your comment. We revised the following part (new text has been italicized): “People without jobs and low education have less hypertension knowledge, making them priority groups for tailored education on health care level as well as community level, lowering the burden of hypertension. *Almost half of the hypertensive patients did not take their medicines* and therefore, adherence to treatment of hypertension should be an important element for future health education *.”*
